# UiO-66 Selective Enrichment Integrated with Thermal Desorption GC-MS for Detection of Benzene Homologues in Ambient Air

**DOI:** 10.1155/2021/3138436

**Published:** 2021-12-14

**Authors:** Xing-Tao Lin, Ge Sun, Jing-Qiang Zhao, Ling-Li Tang, Sheng-Hua Li, Ya-Bo Xie

**Affiliations:** ^1^Faculty of Environment and Life, Beijing University of Technology, Beijing 100124, China; ^2^School of Materials Science and Engineering, Beijing Institute of Technology, Beijing 100081, China

## Abstract

In this study, UiO-66 was selected as sorbent media packed in the tube to selectively enrich trace levels of benzene homologues such as benzene, toluene, and xylene (BTX) in ambient air prior to thermal desorption (TD)-GC-MS determination. A series of experiments were conducted to obtain the optimal TD conditions. The results indicated that the optimal TD parameters were as follows: desorption temperature of 180°C, desorption flow rate of 50 mL min^−1^, and desorption time of 30 min. Furthermore, the method based on UiO-66 enrichment integrated with TD-GC-MS for trace levels of BTX was successfully developed. It exhibited a good linearity (*R*^2^ > 0.99) in the range of 50–1000 ng, except for p, m-xylene in the range of 100–2000 ng, and achieved the recovery of 69.4–101.3%, and the relative standard deviation of 3.8–6.4%. The detection limits of BTX were 1.6–4.0 ng; according to 10 L of sampling volume, the method detection limits would be in the range of 0.16–0.40 *µ*g m^−3^. Additionally, the method was successfully applied to determine BTX in indoor air and showed good selectivity and sensitivity. In summary, the findings in this work revealed that UiO-66 was an attractive adsorbent for selective enrichment trace levels of BTX compounds in ambient air, which was favorable for the subsequent detection by TD-GC-MS.

## 1. Introduction

Up to date, benzene homologues, such as benzene, toluene, and xylene (BTX), are regarded as one of the main compositions of volatile organic compounds (VOCs) in air [1]. BTX pollutants, not only coming from petrol application but from the decoration materials, are detrimental to human health [2–4]. Particularly, benzene has been classified as a carcinogen by the World Health Organization because of its high toxicity and prevalence in air [5]. Hence, lots of regulations and guidelines have already been established to control the BTX pollution [6, 7].

BTX compounds, being present in air at trace level (ppb), are commonly collected using commercial sorbent tubes with the active sampling method and subsequently be transferred into a thermal desorption (TD) system connected to a GC or GC-MS setup. It is well accepted that the sorbent tube sampling method is one of the most widespread approaches to collect and determine the gaseous pollutants like VOCs in ambient air. Tenax TA [8] and activated carbon [9] are the widely used sorbents for VOCs sampling in air, following TD due to their high thermal stability. Although Tenax TA is a type of porous polymers with low background signals, it is not suitable for sampling compounds with high volatility, especially for those boiling point <100°C, because of its low specific surface area (35 m^2^ g^−1^) [10]. Activated carbon generally possesses the large specific surface area, but its hydrophilic features make water more strongly retained on the surface, thereby inhibiting its application in high humidity environment [11]. Thus, it is necessary to develop a new and efficient adsorption material with large surface area, hydrophobic property, and high thermal stability to enrich BTX in air.

Metal-organic frameworks (MOFs) are a novel type of nanoporous materials constructed from metal ions (or clusters) interconnected by organic ligands. Indeed, MOFs materials have gained more and more attention due to their large surface area, tunable pore structure, and good thermal and chemical stability [12, 13]. To date, MOFs have been widely applied in various fields including separation, gas adsorption, and catalysis [14–18]. Typically, some MOFs nanomaterials with large surface area and high thermal stability are hydrophobic and show great potential as efficient sorbents for VOCs. In the past few years, great efforts have been conducted to depict the excellent adsorption capacity of MOFs against VOCs [19–23].

However, most research have been studied just at high pressure or high concentration of pollutants instead of ambient conditions where air pollutants are at ppb level. The adsorption capability for pollutants rapidly decreased with the decrease of pressure and concentration [24]. Thus, it would be more objective to evaluate the sorbent performance in real world application. Recently, some studies have focused on the application of MOFs to adsorb and detect low levels of pollutants. For instance, Zhao et al. [25] used ZIF-7 as sorbent to measure VOCs in ambient air. MOF-5 was also selected as sorbent to determine ppb level of formaldehyde as reported by Kim [26] and Gu [27].

Herein, UiO-66, a typical kind of MOFs, exhibits the positive features such as high thermal stability, hydrophobicity, and large specific surface area, which are critical for the efficient adsorption of air pollutants [28, 29]. In addition, UiO-66 crystal structure consists of octahedral (1.1 nm in diameter) and tetrahedral cavities (0.8 nm in diameter), whose sizes are slightly larger than the molecular sizes of BTX [30]. Furthermore, both of UiO-66 and benzene homologues contain the benzene rings, thereby, resulting in the interaction between the benzene rings in UiO-66 and BTX. The above excellent features of UiO-66 and the similarity in UiO-66 and BTX indicates that UiO-66 could be considered for a sorbent packed in tubes that are compatible with thermal desorption. Therefore, in this work, UiO-66 with typical structure was synthesized and used as a sorbent packed in the tube to enrich the trace BTX in ambient air. In addition, a novel method based on the UiO-66 sorbent tube in combination with TD-GC-MS for BTX determination was developed and successfully applied to ambient air.

## 2. Experimental

### 2.1. Materials and Chemicals

The standard mixture of benzene homologues (1000 mg L^−1^ of each compound in methanol) containing benzene, toluene, p-xylene, m-xylene, and o-xylene (BTX) was purchased from Macklin Biochemical Technology Co. Ltd. (Shanghai, China). The working standard solutions were prepared by diluting the standard mixture with methanol at different concentration levels (50, 100, 200, and 500 mg L^−1^) in 2 mL vials. The carrier gas of helium (99.999%) was purchased from Beijing Praxair Gas Plant (Beijing, China). Zirconium tetrachloride and terephthalic acid were purchased from J&K Scientific Ltd. (Beijing, China). Hydrochloric acid, N, N′-dimethylformamide (DMF), methanol, and acetone were purchased from National Medicine Group Shanghai Chemical Reagent Company (Shanghai, China).

### 2.2. Instrumentation

Field-emission scanning electron microscope (FE-SEM) analysis was conducted on a S-4300 SEM (10 kV) (Hitachi, Japan). Samples were treated via Au sputtering before observation. The powder X-ray diffraction pattern (PXRD) of UiO-66 was recorded with a SmartLab 3 X-ray powder diffractometer (Rigaku, Japan) equipped with a Cu-sealed tube (*λ* = 1.54178 Å). FT-IR spectra were collected on the Nicolet 380 Fourier transform infrared spectrophotometer (Thermo Fisher, U.S.) in the range of 400–4000 cm^−1^. Nitrogen adsorption and desorption isotherms and pore sizes were measured using an Autosorb-iQ specific surface and pore analysis instrument (Quantachrome Instruments, U.S.) operated at 77 K. The thermal stability of UiO-66 was evaluated on a TGA/DSC 3+ thermogravimetric analyzer (Mettler Toledo, Switzerland) from 30°C to 600°C with a ramping rate of 10°C min^−1^ under N_2_ atmosphere. A TurboMatrix 350 TD (Perkin Elmer, U.S.) instrument coupled with a Clarus 600 GC-MS (Perkin Elmer, U.S.) was used to determine BTX in ambient air enriched by the UiO-66 sorbent tube.

### 2.3. Sorbent Tubes Based on UiO-66

UiO-66 was synthesized according to the procedures in literature [31] with slight modification. A mixture containing 930 mg zirconium tetrachloride, 1320 mg terephthalic acid, and 0.67 mL concentrated hydrochloric acid was dissolved into 24 mL DMF in a Teflon-lined bomb. After ultrasonic dispersion for 5 min, the bomb was sealed and placed in an oven at 150°C for 24 h. After cooled down to room temperature, the precipitate was filtered and thoroughly washed with DMF (3 × 15 mL) and acetone (3 × 15 mL), and UiO-66 nanomaterial was obtained after vacuum drying at 80°C.

To reduce the pressure in the tube caused by the fine powder UiO-66, UiO-66 (10 mg) was mixed with 102 acid-washed white support (100 mg, 60–80 mesh, Tianjin Boruijianhe Chromatography Technology Co., Ltd., China). The mixture was placed in a glass tube (6.35 mm × 90 mm, Perkin Elmer, U.S.), plugged with 20 mg glass wool on both of the ends. Subsequently, UiO-66-based sorbent tubes were activated at 250°C for 30 min under a N_2_ flow (100 mL min^−1^). The activation process might be repeated if there was an obvious response in chromatogram. After activation, UiO-66-based sorbent tubes were sealed with end caps and stored in a glass dryer for the follow-up application.

### 2.4. TD-GC-MS Analytical Process

**Figure 1:**
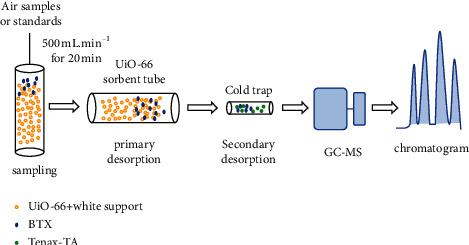
Scheme of analytical procedure on the UiO-66 sorbent tube.

First, working standard solution (1.0 *µ*L) was spiked into the sorbent tube using a GC syringe (SGE Analytical Science, Australia). A pure N_2_ flow (500 mL min^−1^) passed through the sorbent tube for 20 min at room temperature to remove the solvent in the tube and simulate the sampling process. Then, the sorbent tube was placed in the TD system and thermally desorbed at the flow rate of 50 mL min^−1^ for 30 min at 180°C. The eluted gas was reconcentrated on a cold trap (combination of glass wool and Tenax TA) at −10°C and further desorbed at 300°C for 5 min to come into the GC-MD system for analysis (Figure 1).

Five targeted BTX compounds were separated using a HP-5 MS column (30 m length, 0.25 mm i.d., 0.25 *μ*m thickness, Agilent, USA) for detection by GC-MS. The parameters of the GC-MS system are given in Table 1. SIM mode was used for the quantitation of the analytes.

## 3. Results and Discussion

### 3.1. Characterization of UiO-66

**Figure 2:**
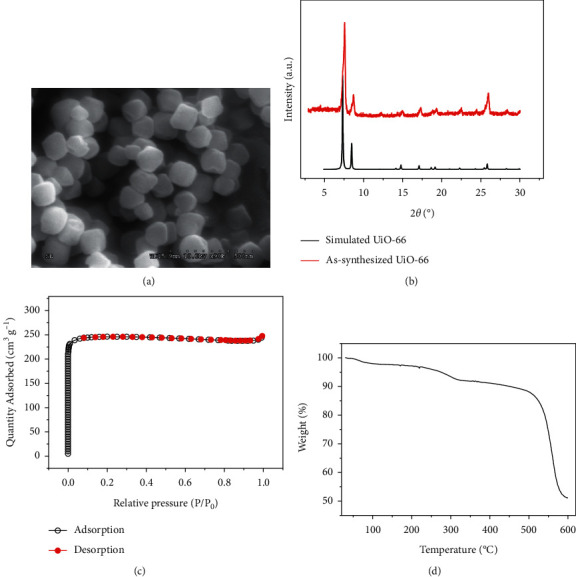
Characterization of UiO-66. (a) SEM image. (b) XRD patterns of simulated and as-synthesized UiO-66. (c) Nitrogen adsorption/desorption isotherms. (d) TGA curve.

Figure 2(a) shows the SEM morphology of the synthesized UiO-66. The UiO-66 had a uniform size and was dispersed evenly. The size of the UiO-66 particles was approximately 100–150 nm, which is slightly smaller than the size that has been reported [32]. The crystallinity of the synthesized UiO-66 was characterized by XRD patterns. As shown in Figure 2(b), the diffraction angles and intensities of the peaks in the synthesized UiO-66 were consistent with the simulated results. This evidenced the successful preparation of UiO-66. FT-IR spectrum is shown in Figure S1. The peaks at 1590 and 1390 cm^−1^ corresponded to the stretching vibrations of the carboxylate groups, and the bands at 729 and 681 cm^−1^ would be assigned to the stretching vibrations of Zr–O. Interestingly, the results were in good line with data reported in the previous literature [28]. N_2_ adsorption-desorption isotherm of UiO-66 is shown in Figure 2(c). It was obvious that the isotherm was typical type I profile. The calculated Brunauer-Emmett-Teller (BET) surface area of UiO-66 was as high as 1010.5 m^2^ g^−1^, and the average pore size was 1.5 nm, which was also consistent with the previous reports [33]. TGA curve shown in Figure 2(d) presents that the weight loss was only 5% at 250°C and was less than 15% even at 520°C. It demonstrated the good thermal stability of UiO-66 at the operating temperatures in TD-GC-MS, as elucidated by Zhao et al. [30].

### 3.2. Optimization of Thermal Desorption Conditions

To get rid of the influence of the white support for BTX adsorption, both a UiO-66-based tube (a mixture of 10 mg of UiO-66 and 100 mg of white support) and a white support tube (only 110 mg of white support) were prepared. The same volumes of analytes were spiked into the two tubes and a N_2_ flow (500 mL min^−1^) passed through for 20 min. Then, the detection was conducted by TD-GC-MS. The results indicated that the peak areas of analytes from white support tube were below 5% of that from the UiO-66 tube. It demonstrated that the adsorption capacity of white support was negligible, and UiO-66 played the critical role in pollutant adsorption.

After enrichment on the UiO-66 tube, analytes were desorbed by thermal desorption. Generally, the efficiency of desorption is great important for the analytical procedure. Typically, thermal desorption was carried out in two steps. In the first step, known as primary desorption, analytes were desorbed and carried into the cold trap by helium gas where analytes were enriched at extremely low temperature. In the second step, known as secondary desorption, the trapped compounds were desorbed by quickly heating the cold trap and transferred into the capillary column for analysis. Indeed, the primary desorption process was critical to the accuracy of the analytical method. Several parameters including desorption temperature, desorption flow rate, and desorption time greatly affected the desorption efficiency in the primary desorption process. Therefore, the primary desorption parameters such as desorption temperature (120, 150, and 180°C), desorption time (10, 20, and 30 min) and desorption flow (10, 30, and 50 mL min^−1^) were optimized with orthogonal experimental design on a UiO-66 sorbent tube loaded with 1000 ng of each BTX compounds.

**Figure 3:**
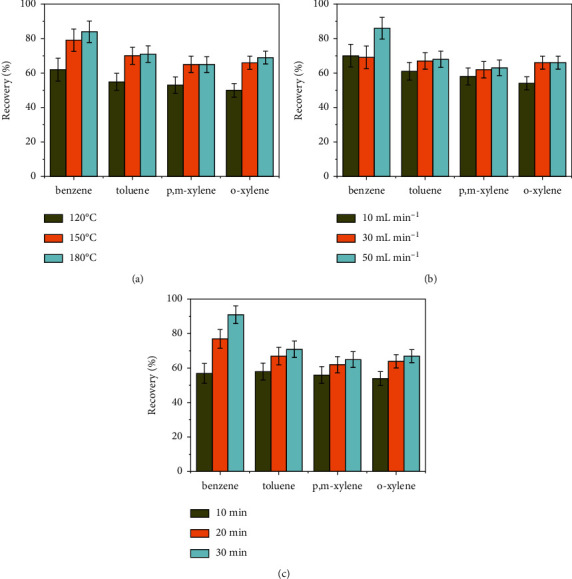
Effect of (a) desorption temperature, (b) desorption flow rate, and (c) desorption time, on desorption efficiency of BTX.

The effect of desorption temperature is shown in Figure 3(a). In all cases, the recovery efficiencies of the analytes at 150°C were obviously higher than that at 120°C. For benzene and o-xylene, the desorption efficiency at 180°C was the highest. On the contrary, no significant change was noted for toluene and p, m-xylene between 150°C and 180°C. Therefore, the desorption temperature was set at 180°C.

The desorption flow rate is one of the key factors in thermal desorption. It should be high enough to completely desorb the targeted compounds. In contrast, the superhigh value might be detrimental to efficiently trap the compounds in the cold trap. Herein, different desorption flow rates (10, 30, and 50 mL min^−1^) were selected in the orthogonal experiments. As shown in Figure 3(b), no obvious difference was noted for toluene and p, m, o-xylene in the range of 30 and 50 mL min^−1^. However, benzene exhibited higher recovery at the flow rate of 50 mL min^−1^. Therefore, the flow rate of 50 mL min^−1^ was used to ensure the maximum desorption of these compounds.

To determine the optimal desorption time, the desorption time from 10 to 30 min was investigated. As shown in Figure 3(c), the longer desorption time corresponded to the higher recoveries in all cases. To further evaluate the effect on desorption efficiency, the desorption time was prolonged to 40 min at the optimal desorption temperature and flow rate. Only a slight improvement of recovery occurred upon increasing the desorption time to 40 min. Considering the time-cost, 30 min was selected for the posttests.

### 3.3. Method Evaluation

For the calibration analysis of BTX, 1.0 *μ*L of each level of working standard liquid (50, 100, 200, 500, and 1000 mg L^−1^) was spiked into UiO-66 sorbent tubes, simulating the sampling process just mentioned in Section 2.4. To compare the sensitivity of the method based on UiO-66 and Tenax TA, the calibration analysis of Tenax TA was also done. After TD-GC-MS analysis, all calibration curves of BTX and Tenax TA depicted the excellent linearity (*R*^2^ > 0.99) in the range of 50–1000 ng (Figure S2), except for p, m-xylene in the range of 100–2000 ng. Unfortunately, p-xylene and m-xylene cannot be separated in our GC method. The calibration curves of benzene, toluene, p, m-xylene, and o-xylene gained from UiO-66 exhibited higher slope than that from Tenax TA. It indicated that the UiO-66 tube presented the better sensitivity than Tenax TA.

The method detection limit (MDL) was calculated according to U.S.EPA guidelines as standard deviation (SD) of seven replicates of a low concentration multiplied by the value 3.14 [34]. The MDL of BTX was between 1.6 and 4.0 ng (Table 2).

Assuming an air sampling volume of 10 L, the corresponding MLD value was in the range of 0.16–0.40 *μ*g m^−3^. To compare the MDL of BTX based on UiO-66 sorbent with that on Tenax TA sorbent, the MDL of BTX based on Tenax TA sorbent was also done. The MDL of benzene, toluene, p, m-xylene, and o-xylene was 8.3, 9.1, 11.3, and 8.7 ng, respectively. Assuming an air sampling volume of 10 L, the corresponding MLD value based on the Tenax TA sorbent tube was in the range of 0.83–1.13 *μ*g m^−3^. Obviously, the MDL of BTX based on the UiO-66 sorbent tube was lower than that on the Tenax TA sorbent tube. It indicated that the UiO-66 enrichment method developed in this work was suitable to quantify BTX at ppt level in ambient air and possessed better sensitivity than Tenax TA. Zhao et al. [25] used ZIF-7 as sorption media and examined the ZIF-5 method for VOCs determination. It reported that limit of detection (LOD) (S/N = 3) of benzene and toluene was 1.88 and 1.22 ng, respectively. These values were similar with the results in this work. But the amount of ZIF-7 (150 mg) was 15 times that of UiO-66 (10 mg) in this work.

Five replicates loaded with 1 *μ*L of 1000 mg L^−1^ working standard were conducted to determine the repeatability of the TD-GC-MS method. Herein, the relative standard deviation (RSD) was in the range of 3.8%–6.4% (Table 2).

Recovery was measured by the peak area obtained from the analysis of a standard tube by a TD-GC-MS method versus the peak area obtained by directly injecting the same amount of standard into GC-MS. Obviously, the recovery of benzene (101.3%) was higher than that of toluene (77.6%) and xylene (69%) (Table 2). One of reasons for adsorption of BTX by UiO-66 was the pore-filling mechanism [35]. The kinetic diameter of benzene (0.58 nm), toluene (0.67 nm), and xylene (o-xylene: 0.74 nm, p-xylene: 0.67 nm, m-xylene: 0.71 nm) [36] was less than the pore size of UiO-66 (1.5 nm) synthesized in this work. Thus, all of them could diffuse into the porous materials. However, toluene and xylene with larger molecular sizes would interact with more surface atoms on MOFs, which enhance the host-guest interaction [37]. Consequently, it may be difficult for toluene and xylene to desorb from UiO-66 and obtained lower recovery than benzene. In addition, the boiling point of benzene was lower than the others. As a result, it may be easier for benzene to desorb from UiO-66 compared with toluene and xylene.

### 3.4. Tube Breakthrough

To investigate the tube breakthrough, two sorbent tubes were connected in series. The working standard (10–25 *μ*L of 1000 mg L^−1^) was injected into the first sorbent tube. Subsequently, a pure N_2_ flow (500 mL min^−1^) was passed through the two tubes for 20 min and followed by TD-GC-MS determination. When the responses obtained from the second tube reached 5% of the total amount from both tubes, it indicated the occurrence of a breakthrough [38]. When 10.0*μ*L of 1000 mg L^−1^ of each BTX compounds was loaded on the UiO-66-based sorbent tube, only benzene was detected on the second tube with the value of 4.5%. With increasing the volume to 25.0 *μ*L, other compounds including toluene, p, m-xylene, and o-xylene were detected with the concentration below 1%. Therefore, the tube breakthrough point of benzene was 10.0 *μ*g, while the value for the others was more than 25.0 *μ*g. It should be noted that the results in this work were better than the study on ZIF-7 (2.0 *μ*g) [25]. It revealed that UiO-66 might be an excellent adsorbent for BTX compounds.

### 3.5. Recyclability

**Figure 4:**
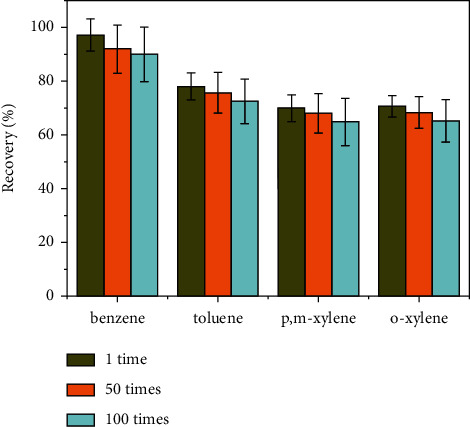
Recyclability of the UiO-66 tube.

It is well considered that good recyclability is an important factor for the sorbent to be applied in separation field. Therefore, the cycle tests were conducted for the UiO-66 packed tube. As shown in Figure 4, the recovery efficiency of BTX did not exhibit significant loss even after 100 times of adsorption and desorption cycles through the TD-GC-MS system with the same UiO-66 packed tube. This finding implied the remarkable recyclability of the UiO-66 sorbent tube.

### 3.6. Application to Real Samples

To verify the feasibility of the method based on UiO-66 sorbent, it was compared with commercial sorbent Tenax TA. Both sorbent tubes were prepared to obtain the same volume of indoor air samples in the same locations. Both sorbent tubes were connected with active pump samplers at the flow rate of 500 mL min^−1^ for 20 min and analyzed under their own optimal TD-GC-MS conditions (TD conditions for Tenax TA: desorption temperature was 250°C, desorption time was 20 min, desorption flow rate was 50 mL min^−1^). TD conditions for UiO-66 followed with the optimal parameters as determined in the above section. The concentrations of benzene, toluene, and xylene in indoor air samples analyzed by two methods based on the different sorbent tubes were similar as given in Table 3, thereby, demonstrating the accuracy and reliability of the method based on UiO-66 sorbent tube.

**Figure 5:**
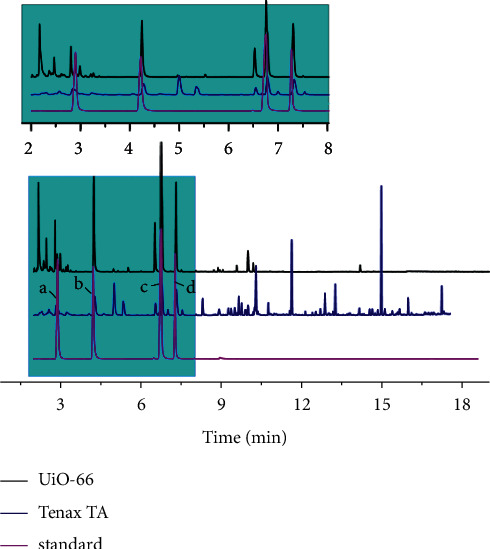
Chromatograms of samples of standard, enrichment on Tenax TA and UiO-66: (a) benzene; (b) toluene; (c) p, m-xylene; (d) o-xylene.

Comparing the chromatogram of UiO-66 to that of Tenax TA (Figure 5), it was obvious that in the chromatogram of Tenax TA, more peaks appeared after the time of BTX (8 min), indicating more impurities with higher boiling points than BTX were captured. The finding evidenced that Tenax TA might be suitable for compounds with relatively high boiling points, but not an appropriate absorbent for BTX [39]. On the contrary, less peaks observed in the chromatogram of UiO-66 demonstrated that UiO-66 was more suitable for those VOCs with relatively low boiling points and high volatility, such as benzene, toluene, and xylene, especially for benzene. The less impurity peaks and higher peak height which appeared in the chromatogram of the UiO-66 sorbent tube showed that UiO-66 possessed the good selectivity and sensitivity for BTX. It might be interpreted by the fact that the molecular sizes of adsorbates (e.g., benzene, toluene, and xylene) were smaller than the pore size of UiO-66. It was also considered that the *π*-*π* interaction of benzene homologues with the metal centers and benzene rings in the linkers of UiO-66 was responsible for the high selectivity [40]. UiO-66 with higher slope of calibration curves and lower MDL of BTX than Tenax TA further demonstrated its good sensitivity.

## 4. Conclusions

A method based on the UiO-66 sorbent tube followed by TD-GC-MS for determination of BTX in air was developed. Typically, the feasibility of the utilization of UiO-66 as a sorbent to sample and enrich BTX in ambient air has been evidenced. Furthermore, the UiO-66-based detection approach exhibited the higher selectivity and sensitivity for BTX compounds than the method relied on commercial adsorbent Tenax TA, indicating that the developed method presented the potential practical application in selective enrichment of BTX. The findings in this work might shed light on the large-scale application of MOFs nanomaterials as adsorbents for air pollutants.

## Figures and Tables

**Table 1:** Operating conditions of TD-GC-MS for BTX analysis.

TD method	GC-MS method
Sampling tube: mixture of UiO-66 and white support	GC method column: HP-5 MS (30 m × 0.25 mm × 0.25 *μ*m);Initial temp.: 50°C
Desorption temp.: 180°C	Initial hold time: 5 min
Desorption time: 30 min	Oven rate: 10°C min^−1^
Desorption flow rate: 50 mL min^−1^	Final temp.: 250 °C Final hold time: 5 min
Cold trap: Tenax TA (100 mm × 3.2 mm)	MS method Ionization mode: EI (70 eV)
Adsorption temp.: −10°C	Ion source temp.: 250°C
Desorption temp.: 300°C	Transfer line temp.: 250°C
Desorption time: 5 min	TIC scan range: 50–500 m/*z*
Column flow rate: 1 mL min^−1^	Selected ions Benzene: 78
Split ratio: 9	Toluene: 91, 92
Transfer line temp.: 250°C	*o*, p, m-Xylene: 91, 106
Valve temp.: 230°C	

**Table 2:** Method evaluation of BTX.

Compound	Recovery (%)	RSD (%) (*n* = 5)	MDL (ng)	MDL (*μ*g m^−3^)
Benzene	101.3	6.4	2.0	0.20
Toluene	77.6	4.9	1.6	0.16
p, m-Xylene	69.8	4.7	4.0	0.40
o-Xylene	69.4	3.8	2.4	0.24

**Table 3:** Comparison of BTX concentrations in indoor air samples analyzed with different sorbent tubes (*μ*g m^−3^).

Sorbent tube	Location	Benzene	Toluene	p, m-Xylene	o-Xylene
UiO-66	Bedroom	13.0	15.7	24.0	20.8
	Living room	13.3	15.2	23.9	20.3

Tenax TA	Bedroom	16.7	15.4	27.6	22.6
	Living room	17.9	15.2	25.4	21.3

## Data Availability

The data used to support the findings of this study are included within the article.

## Supplementary Materials

Supplementary Materials Figure S1. FT-IR spectra of UiO-66. Figure S2. The calibration curves of (a) benzene, (b) toluene, (c) p, m-xylene, and (d) o-xylene obtained from Tenax TA and UiO-66.
