# Supplementary material for: UiO-66 Selective Enrichment Integrated with Thermal Desorption GC-MS for Detection of Benzene Homologues in Ambient Air
Source: J Anal Methods Chem. 2021 Dec 14;2021:3138436. doi: 10.1155/2021/3138436 (PMC8692002; doi:10.1155/2021/3138436)
Supplement: Supplementary Materials — Figure S1. FT-IR spectra of UiO-66. Figure S2. The calibration curves of (a) benzene, (b) toluene, (c) p, m-xylene, and (d) o-xylene obtained from Tenax TA and UiO-66. [file 3138436.f1.docx]

Supplementary Materials

**UiO-66 selective enrichment integrated with thermal desorption GC-MS for detection of benzene homologues in ambient air**

**Xing-Tao Lin,^1^ Ge Sun,^1^ Jing-Qiang Zhao,^1^ Ling-Li Tang,^1^ Sheng-Hua Li,^2^ and Ya-Bo Xie*^1^**

^1^ Faculty of Environment and life, Beijing University of Technology, Beijing 100124, China

^2^ School of Materials Science and Engineering, Beijing Institute of Technology, Beijing 100081, China

Correspondence should be addressed to Ya-Bo Xie; [Xieyabo@bjut.edu.cn](mailto:Xieyabo@bjut.edu.cn)


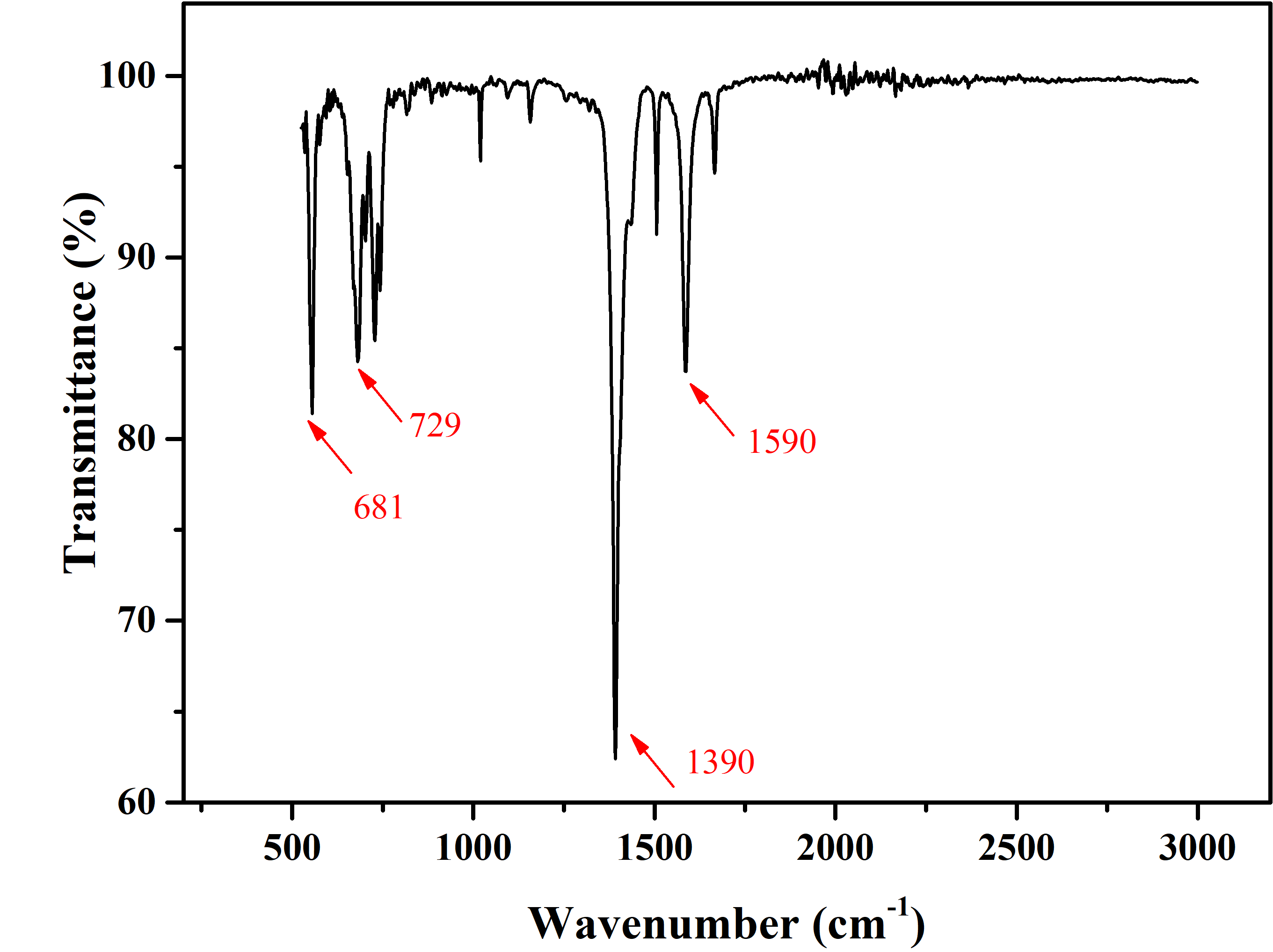


FIGURE S1: FT-IR spectra of UiO-66

| 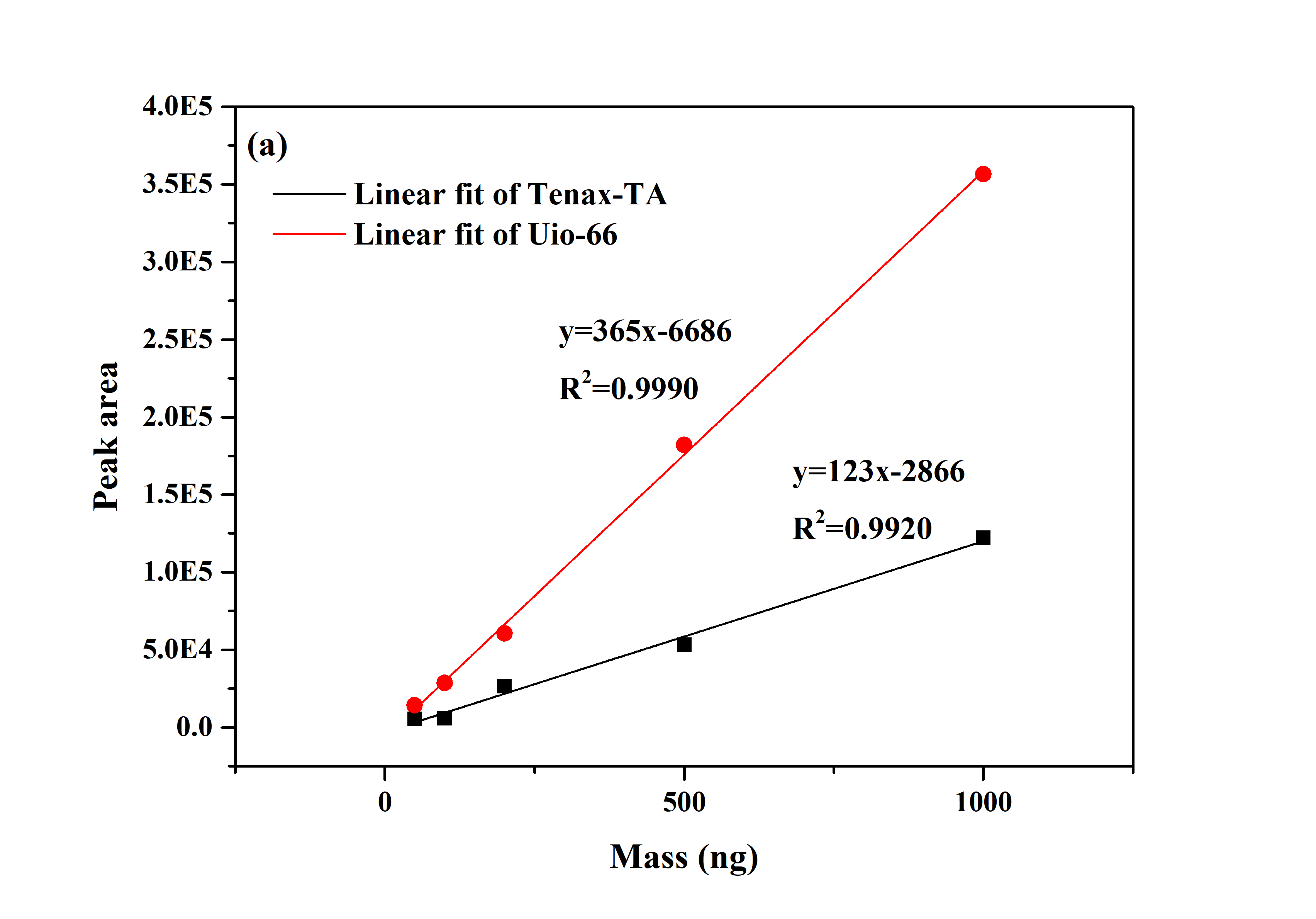 | 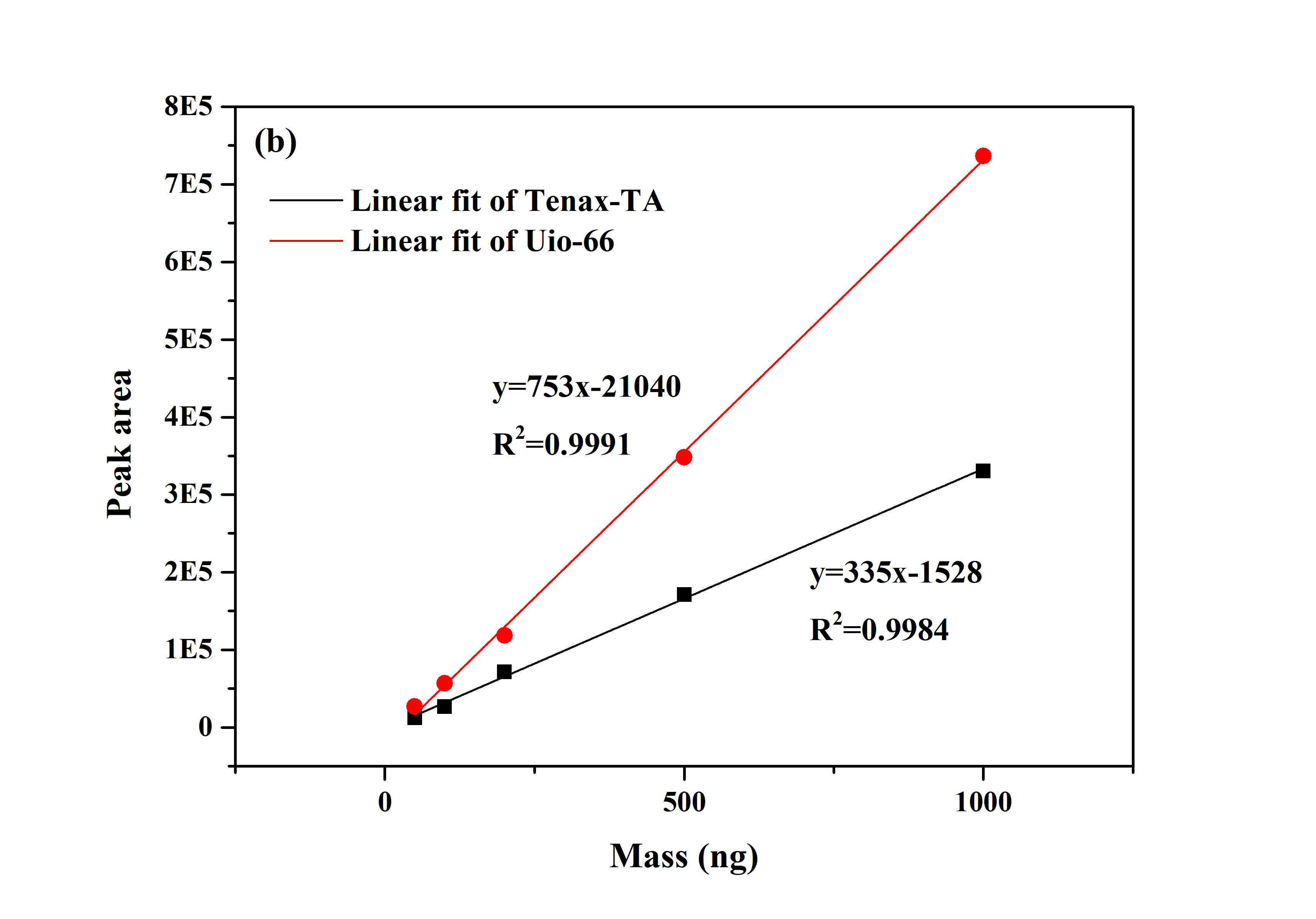 |
| --- | --- |
| 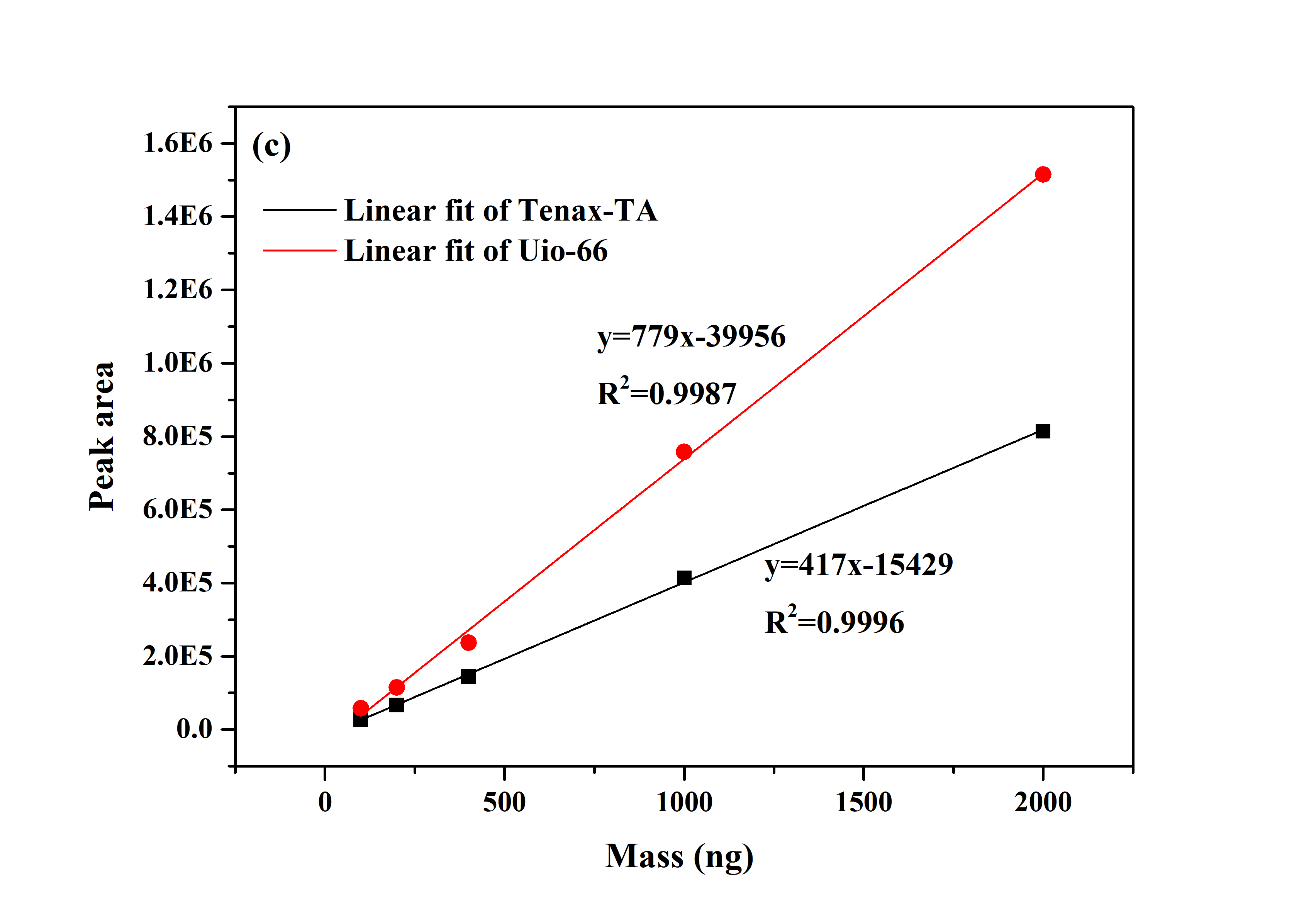 | 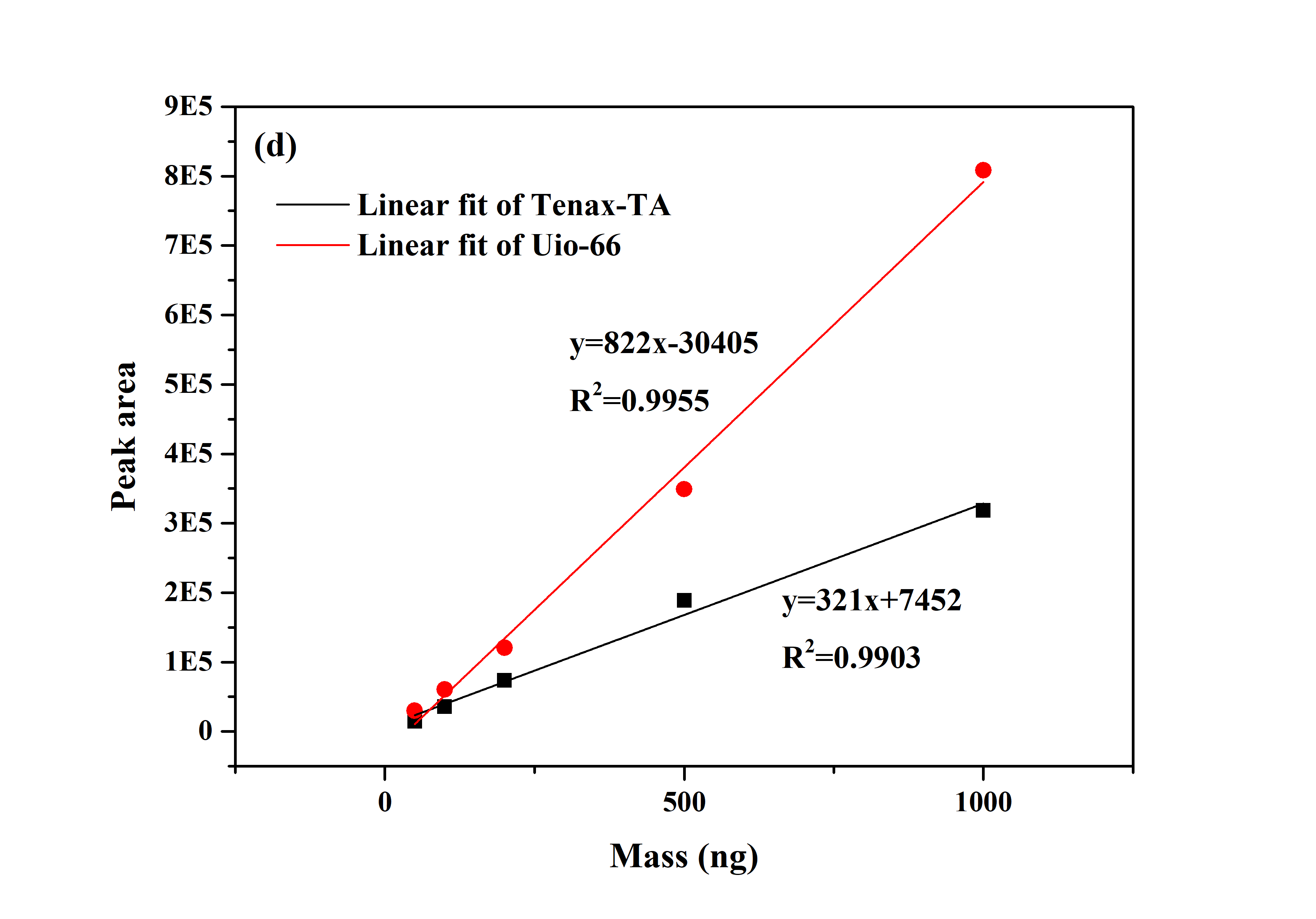 |

FIGURE S2: The calibration curves of (a) benzene, (b) toluene, (c) p, m-xylene, (d) o-xylene obtained from Tenax-TA and UiO-66.
